# Microbial Community Successional Changes in a Full-Scale Mesophilic Anaerobic Digester from the Start-Up to the Steady-State Conditions

**DOI:** 10.3390/microorganisms9122581

**Published:** 2021-12-13

**Authors:** Barbara Tonanzi, Simona Crognale, Andrea Gianico, Stefano Della Sala, Paola Miana, Maria Chiara Zaccone, Simona Rossetti

**Affiliations:** 1 National Research Council of Italy Water Research Institute CNR-IRSA, Area della Ricerca RM1, Monterotondo, 00015 Rome, Italy; simona.crognale@irsa.cnr.it (S.C.); andrea.gianico@irsa.cnr.it (A.G.); simona.rossetti@irsa.cnr.it (S.R.); 2 Veritas S.p.a., 30135 Venezia, Italy; s.dellasala@gruppoveritas.it (S.D.S.); p.miana@gruppoveritas.it (P.M.); c.zaccone@gruppoveritas.it (M.C.Z.)

**Keywords:** waste-activated sludge, full-scale plant, anaerobic digestion, 16S rRNA gene sequencing, microbial population dynamics

## Abstract

Anaerobic digestion is a widely used technology for sewage sludge stabilization and biogas production. Although the structure and composition of the microbial communities responsible for the process in full-scale anaerobic digesters have been investigated, little is known about the microbial successional dynamics during the start-up phase and the response to variations occurring in such systems under real operating conditions. In this study, bacterial and archaeal population dynamics of a full-scale mesophilic digester treating activated sludge were investigated for the first time from the start-up, performed without adding external inoculum, to steady-state operation. High-throughput 16S rRNA gene sequencing was used to describe the microbiome evolution. The large majority of the reads were affiliated to fermentative bacteria. *Bacteroidetes* increased over time, reaching 22% of the total sequences. Furthermore, *Methanosaeta* represented the most abundant methanogenic component. The specific quantitative data generated by real-time PCR indicated an enrichment of bacteria and methanogens once the steady state was reached. The analysis allowed evaluation of the microbial components more susceptible to the shift from aerobic to anaerobic conditions and estimation of the microbial components growing or declining in the system. Additionally, activated sludge was investigated to evaluate the microbial core selected by the WWTP operative conditions.

## 1. Introduction

Anaerobic digestion (AD) is a well-known technology that uses mixed microbial communities for the treatment of a wide range of organic waste streams and subsequent conversion to biogas [1]. In fact, due to its potential impacts on the environment and human health, sewage sludge requires proper treatment and management. Among organic streams, AD can be efficiently applied for the valorization of waste-activated sludge (WAS) due to its cost-effectiveness and resource recovery capacities [2]. The treatment and disposal of the excess sludge produced by wastewater treatment plants (WWTPs) is a serious environmental problem and represent up to 50% of the operating costs of the plant [3]. AD is a multistage bioprocess essentially covered by four sequential stages (hydrolysis, fermentation, acetogenesis, and methanogenesis), which can reduce the amount of organic matter up to 50%. During the AD process, organic matter is degraded to biogas (rich in methane, CH_4_), water, ammonia, and other mineral compounds. The biogas produced through the AD process has a high calorific value and is considered as a renewable energy source [4]. The characterization of the microbial communities involved in the different AD stages, and especially of the key players and associated metabolic pathways, is one of the most important objectives to be achieved in order to fully understand and effectively manage the process [5,6].

Currently, the structure and composition of the microbial communities in a full-scale anaerobic digester have been extensively investigated [3,7,8,9]. However, little is known about the microbial successional dynamics during the start-up phase and how microorganisms respond to variations occurring in such systems under real operating conditions. The microbial communities can be considered unique in each digester mainly due to the different operating conditions and substrate composition [10,11,12]. From this point of view, the identification of a core microbiome and/or marker populations in a full-scale anaerobic digester can be very helpful to estimate the potential metabolic capacity and to guarantee the resilience of the system, key points for the process management [1].

Different groups of microorganisms are involved in the degradation of organic matter and a close interaction among them is essential for the process stability [13,14]. Hence, the anaerobic digesters are particularly susceptible to failure during the start-up phase, especially when easily biodegradable substrates are applied [15,16,17].

In particular, the development of an active microbial biomass that achieves an adequate treatment performance is one of the key points for the success of the AD process [16,18]. The methanogenic activity, carried out by the archaea, could be a limiting step of the AD process [17], since these microorganisms are characterized by slow growth rates and a high sensitivity to different environmental conditions [4]. Thus, the process inhibition could often be due to an imbalance between acid-producing bacteria and methanogens [19].

Generally, the inoculum used for the start-up of a new digester is a consortium obtained from several sources, such as rumen and animal manures, soil extract, or anaerobic sludge from municipal or industrial WWTPs. Among them, the digested sludge taken from a working digester has been proved as a good inoculum for a stable start-up phase [15].

It has been reported that start-up without external inoculum caused significant accumulation of volatile fatty acids (VFAs) and took longer for the organic loading rate to be raised during the early days of operation [20].

The suitable use of aerobic waste-activated sludge (WAS) as a seed source for anaerobic digestion is mainly due to the growth of methanogenic archaea in the anaerobic niches occurring in the sludge flocs [21], since high concentrations of facultative anaerobes bacteria in WAS have been proved [22]. Wu et al. [9] demonstrated that the use of WAS as internal inoculum could be a good option, because it contains a considerable number of methanogenic archaea, and it can be easily taken from WWTPs. Moreover, WAS has a significant capacity to anaerobically biodegrade acetate and propionate at the mesophilic temperature, and after a short acclimatization period, this inoculum could quickly reach high methane yields [21].

Most previous studies focused on the start-up of the anaerobic digester inoculated with WAS carried out in batch or in lab-scale systems and in some cases, they showed that VFAs accumulation and pH decrease can affect the AD process and efficiency [15].

Even though the start-up phase has been extensively studied for lab-scale anaerobic reactors [23,24], few studies have dealt with the microbial community dynamics during the start-up stage in full-scale digesters [16,17,25].

This study analyzed the start-up phase of a full-scale mesophilic anaerobic digester treating municipal WAS, with the aim of describing the microbial population dynamics until the establishment of a steady-state operation, evaluating the process robustness and reliability in a full-scale system when only WAS is used as the seed source.

The digester performance and microbial population dynamics occurring from the start-up phase to the operation under steady-state conditions were investigated for one year. The correlations between the core microbiome, the operational conditions, and the digester performances were also evaluated to determine whether cooperation and dynamics influenced biogas production.

## 2. Materials and Methods

### 2.1. Full-Scale AD Plant

The full-scale AD plant of Fusina (3300 m^3^, Venice, Italy) treats sewage sludge from a municipal wastewater treatment plant (WWTP) designed for 400,000 population equivalent (PE) and serving ~300,000 PE with an average influent flow of 1.1 m^3^/s.

The WWTP influent is characterized by a total suspended solids (TSS) content of 179 ± 63 mg/L, COD of 313 ± 94 mg/L, and BOD_5_ of 160 ± 36 mg/L (BOD_5_/COD = 53 ± 13%). The wastewater treatment line is composed of the following: grid screens, grit and grease removal, pre-denitrification, biological treatment, secondary sedimentation, and final disinfection (UV lamps). The sludge treatment line consists of dynamic pre-thickening, mesophilic anaerobic digestion, post-thickening, and final dewatering.

The sludge flow rate is split between two anaerobic digesters, with a volume of 3300 m^3^ each. The start-up phase was operated under mesophilic conditions by filling the digesters with the WAS produced at Fusina WWTP, by promoting the anaerobic self-degradation process through WAS microbial community shift, without providing external seed sources.

After the filling period (two months), the digesters were operated at the average temperature of 33 ± 4 °C, with a hydraulic retention time (HRT) of about 19 days and a feed sludge amount of ~8 m^3^/h with a total suspended solids (TSS) content of ~5% (volatile suspended solids VSS = 67%TSS). The organic loading rate (OLR) applied was about 1.75 KgSSV/m^3^d.

WAS was sampled and analyzed at four different times during the operation. Digested sludge samples were collected from one of the two anaerobic digesters during one year of operation, immediately stored at −20 °C and used for the microbial analyses.

Alcalinity and volatile suspended solids were measured according to APAT/IRSA 2010.

### 2.2. DNA Extraction

A PowerSoil DNA Isolation kit (MoBio, Carlsbad, CA, USA) was used to extract DNA from 1 mL (~0.25 g wet weight) of digestate or waste-activated sludge samples. DNA was eluted with 50 μL of sterile distilled water and the purity and concentration were verified by a NanoDrop 2000c spectrophotometer (Thermo Scientific, Waltham, MA, USA). Genomic DNA was stored at −20 °C and then used for real-time PCR quantification (qPCR) and high-throughput 16S rRNA gene sequencing.

### 2.3. Real-Time PCR, qPCR

The qPCR absolute quantification assays were performed targeting 16 rRNA genes of the bacteria domain and mcrA genes for methanogens following the detailed procedure reported in Tonanzi et al. [26]. In brief, qPCR reactions for bacteria were performed with TaqMan^®^ chemistry in a 20 μL total volume of SsoAdvancedTM Universal Probes Supermix (Biorad, Milan, Italy), including 3 μL of DNA as template, 300 nM of each primer (Bac1055F and Bac1392R; [27]), and 300 nM of TaqMan^®^ Bac1115 probe (FAM-CAACGAGCGCAACCC-TAMRA; [27]). qPCR reactions targeting mcrA genes were performed with SybrGreen^®^ chemistry in a 20 μL total volume of SsoAdvanced^®^ Universal SYBR^®^ Green Supermix (Biorad, Milan, Italy) including 3 μL of DNA as template and 300 nM of each primer (mlas and mcrA-rev primers; [28]). Standard curves for the absolute quantification were constructed by using the long amplicons method previously reported in Matturro et al. [29]. Each reaction was performed in triplicate with a CFX96 TouchTM Real-Time PCR Detection System (Biorad, Milan, Italy). Quantitative data were expressed as the logarithm of gene copies numbers g^−1^ of volatile suspended solids (VSSs).

### 2.4. High-Throughput 16S rRNA Gene Sequencing

Extracted DNA was amplified in a first PCR with the primer pairs 27F (5′-AGAGTTTGATCCTGGCTCAG-3′) and 534R (5′-ATTACCGCGGCTGCTGG-3′) and 340F (5′-CCCTAHGGGGYGCASCA-3) and 915R (5′-GWGCYCCCCCGYCAATTC-3′) targeting the regions V1–V3 and V3-V5 of bacterial and archaeal 16S rRNA genes, respectively. PCR reactions containing 8–12 ng of DNA were performed by following the protocol described in Crognale et al. [30]. The amplicon libraries were purified using the Agencourt^®^ AMpure XP bead protocol (Beckmann Coulter, Milan, Italy). Sequencing libraries were prepared from the purified amplicon libraries using a second PCR followed by purification. The library concentration was measured with a Qubit 3.0 Fluorometer (Thermo Fisher Scientific, Waltham, MA, USA). The purified libraries were pooled in equimolar concentrations and diluted to 4 nM. The samples were paired end sequenced (2 × 301 bp) on a MiSeq platform (Illumina) using a MiSeq Reagent kit v3, 600 cycles (Illumina, San Diego, CA, USA) following the standard guidelines for preparing and loading samples. A 10% Phix control library was spiked in to overcome the low complexity issue often observed with amplicon samples.

### 2.5. Bioinformatic Processing

After checking read quality with fastqc, the sequences were processed and analyzed using QIIME2 v. 2018.2 [31]. The reads were demultiplexed using demux plugin (https://github.com/qiime2/q2-demux accessed on 10 February 2018) and the primer sequences were removed by using cutadapt plugin (https://github.com/qiime2/q2-cutadapt accessed on 2 December 2017). The demultiplexed reads were denoised, dereplicated, and chimera-filtered using the DADA2 algorithm resolving amplicon sequence variants (ASVs) [32]. Taxonomy was assigned to ASVs using a pre-trained naïve-Bayes classifier based on the 16S rRNA gene database at 99% similarity of the Silva132 release [33]. Rarefaction curves were computed using the Vegan R package.

### 2.6. Statistical Analysis

The principal component analysis (PCA), based on the correlation matrix, was performed by comprising the bacterial and archaeal microbial composition as revealed by 16S rRNA high-throughput sequencing (only families ≥5 % of total reads were considered) using the software PAST (PALAEONTOLOGICAL STATISTICS, ver. 2.17) [34]. All values were normalized by log(X + 1).

## 3. Results

### 3.1. Full-Scale AD Plant

**Figure 1:**
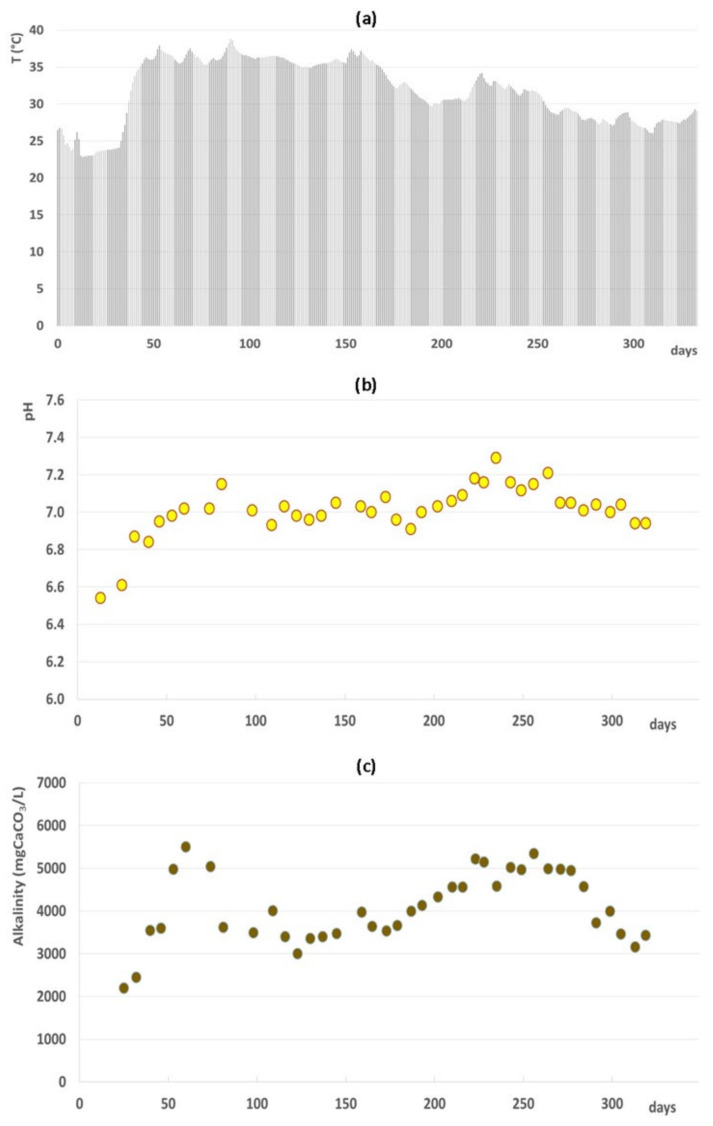
Trend of temperature (**a**), pH (**b**), and alkalinity (**c**) during the first year of operation of the anaerobic digester.

The physical-chemical variations of the full-scale anaerobic digester over 12 months of operation are shown in Figure 1. After the first 40 days of operation, the digester temperature reached stable mesophilic conditions. In parallel, the digester reached high alkalinity levels able to buffer the system and to maintain a neutral pH. Once the steady-state conditions were achieved, the digester maintained stable temperatures (33 ± 4 °C), stable pH (7 ± 0.1), and stable alkalinity levels (4476 ± 660 mgCaCO_3_/L).

**Figure 2:**
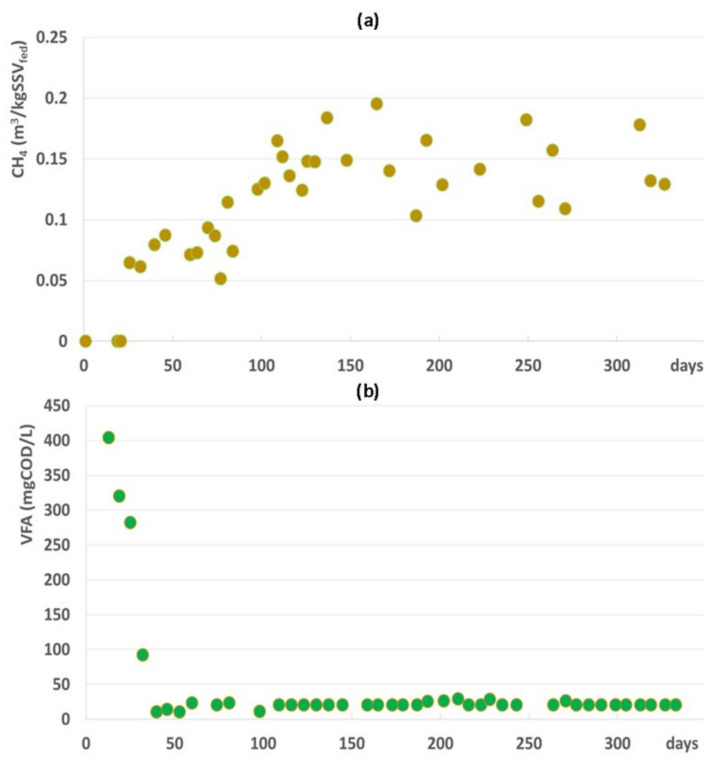
Specific methane production pattern (**a**) and VFA concentration in the digester (**b**).

As regards the digestion performances, scarce methane production was observed during the first 40 days (~2 HRT) of operation (Figure 2a), with a consequent presence of VFA in the digester (Figure 2b). After the first HRT, a rapid consumption of VFA and an increasing trend of the specific methane production occurred, reaching 0.144 ± 0.03 m^3^CH_4_/kgVSS_fed_.

The biogas produced at steady state contained 69 ± 1% methane and was successfully utilized to heat the digester over the first 12 months of operation.

### 3.2. Microbial Composition of Secondary Sludge from Fusina WWTP

**Figure 3:**
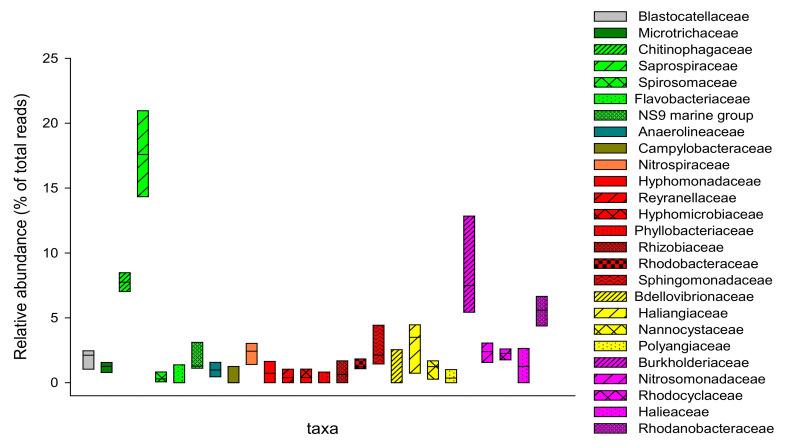
Box plot showing the relative abundance (as % of total reads) of the bacterial families (≥1% of total reads) in secondary sludge sampling along four different periods.

Secondary sludge from Fusina WWTP was collected in four different periods and characterized in order to identify the core microbiome. The findings revealed a similar microbial composition along different sampling periods. In detail, the secondary sludge was characterized by a high abundance of Bacteroidetes (32.0 ± 4%), Proteobacteria (45.2 ± 7%), and Chloroflexi (5.8 ± 3%). Reads affiliated with family Saprospiraceae (17.6 ± 3%) and genera Ferruginibacter (5.2 ± 1%) and Terrimonas (0.7 ± 0.3%) represented the phylum Bacteroidetes (Figure 3). Within the phylum Proteobacteria, Reyranella, Novosphingobium, Pseudorhodobacter, Sphingorhabdus, and Stella comprised the Alphaproteobacteria portion, counting up to 2.1 ± 1% of total reads. Around 5.3 ± 3% of the total reads were affiliated with the families Bdellovibrionaceae, Haliangiaceae, Nannocystaceae Polyangiaceae, and members of the phylum Deltaproteobacteria. The largest portion of Proteobacteria was represented by Burkholderiaceae (8.6 ± 4%), Nitrosomonadaceae (2.3 ± 0.8%), Rhodocyclaceae (2.2 ± 0.4%), and Rhodanobacteraceae (5.5 ± 1%) mainly affiliated with the genera *Acidovorax, Aquabacterium, Ideonella, Leptothrix, Limnohabitans, Rhodoferax, Nitrosomonas, Thauera*, and *Dokdonella*.

### 3.3. Dynamics of Bacterial and Archaeal Communities over the Operation

**Figure 4:**
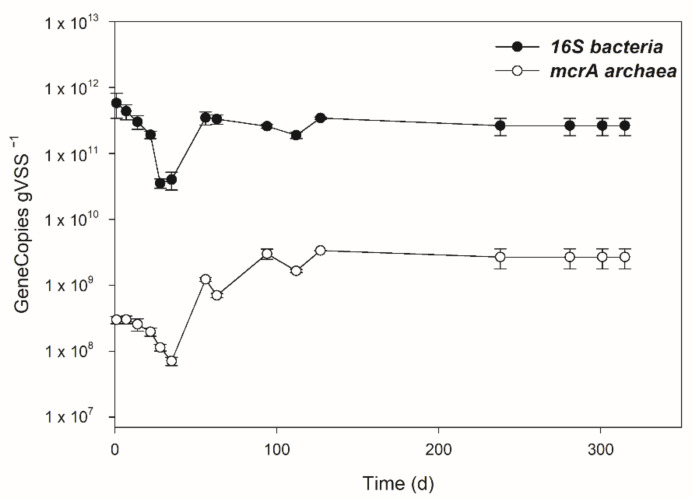
Abundance of methanogens (mcrA gene) and bacteria (16S rRNA gene) estimated by qPCR at different sampling times. Data expressed as gene copies per gram of volatile suspended solid (VSS).

The abundance of total bacteria and methanogens was estimated throughout the operation by qPCR. During the start-up period, the abundance of both microbial components showed a slight decrease to 4.0 *×* 10^10^ ± 1.2 *×* 10^10^ gene copies/gVSS for total bacteria and to 7.1 *×* 10^7^ ± 1.0 *×* 10^7^ gene copies/gVSS for methanogens (Figure 4). After the establishment of steady-state conditions, the analysis revealed that both microbial components were almost constant throughout the plant operation. In particular, bacterial abundance settled around an average value of 2.8 *×* 10^11^ ± 5.1 *×* 10^10^ gene copies/gVSS and methanogens were around 2.3 *×* 10^9^ ± 8.9 *×* 10^8^ gene copies/gVSS (Figure 4).

**Figure 5:**
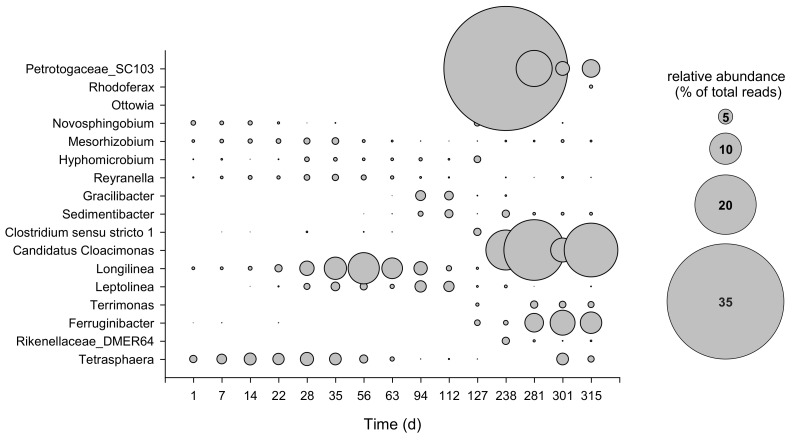
Bubble plot depicting the relative abundance (as % of total reads) of the main bacterial genera (≥2% relative abundance in at least one sample). The bubble size shows the relative abundance.

High-throughput sequencing of the region V1–V3 of the bacterial 16S rRNA gene yielded a total of 288,107 sequence reads after quality control and bioinformatic processing, which resolved into more than 3000 unique sequences. Overall, most of the obtained sequences throughout the whole operation period were mainly affiliated with fermentative bacteria belonging to the phyla *Actinobacteria*, *Bacteroidetes*, *Chloroflexi*, and *Proteobacteria*, followed to a minor extent by *Acidobacteria*, *Firmicutes*, *Patescibacteria*, and *Thermotogae* (Figure S1). *Bacteroidetes* increased over time, representing between 2.4% and 22.5% of the total reads along the whole operation; these reads were mainly affiliated with the family *Saprospiraceae* during the start-up period (0.9–11.3%) and with the families *Prolixibacteraceae* and *Chitinophagaceae*, especially *Ferruginibacter* and *Terrimonas* genera, after steady state was reached (Figure 5). Sequences belonging to the families *Anaerolinaceae* and *Caldilineaceae* of the phylum *Chloroflexi* were observed during the entire operation, with a relative abundance ranging between 1% and 16.5% of the total reads. Among these families, *Leptolinea* and *Longilinea* were the most abundant genera, with, on average, 1.3% and 2.7%, respectively. Within the phylum *Proteobacteria*, *Reyranella*, *Hyphomicrobium*, *Mesorhizobium*, and *Novosphingobium* represented the *Alphaproteobacteria* portion, counting up to 2.1% at day 127 and decreasing to 1.2% at the end of operation (Figure 5). The families *Burkholderiaceae* and *Rhodanobacteraceae*, members of the class *Gammaproteobacteria*, represented up to 24% of the total reads at the end of the operation. *Acidobacteria*, mainly affiliated with the family *Blastocatellaceae*, showed a decrease during the last months of operation, moving from 9.7% of the total reads at day 1 to 3.5% at day 315. *Patescibacteria* was abundant during the start-up (on average 12.1%) and decreased after steady-state conditions were reached, with an average value of 4.6% of the total reads (Figure S1). *Cloacimonetes* and *Thermotogae* showed an increase during the last months of operation with the genus *Candidatus Cloacimonas* and family *Petrotogaceae* representing up to 18.1% and 36.8% of the total reads, respectively, after day 238 (Figure 5 and Figure S1).

**Figure 6:**
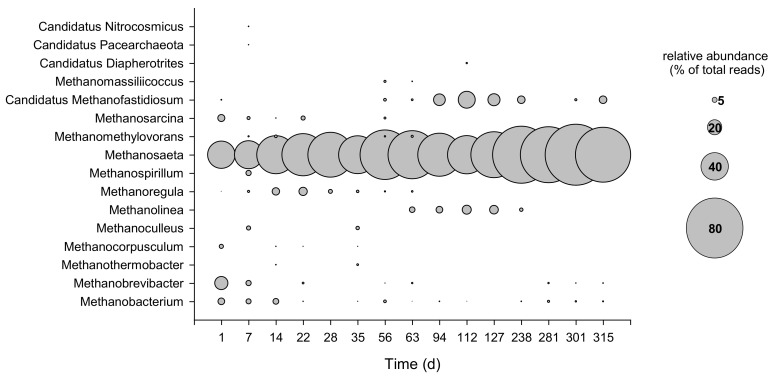
Bubble plot depicting the relative abundance (as % of total reads) of the main archaeal genera (≥1% relative abundance in at least one sample). The bubble size shows the relative abundance.

High-throughput sequencing of the region V3–V5 of the archaeal 16S rRNA gene yielded a total of 270,920 sequence reads after quality control and bioinformatic processing, which resolved into more than 800 ASVs. The phylum *Euryarcheota* represented between 66.8% and 97.7% of the total reads. Sequences affiliated with the family *Methanosaetaceae*, mainly belonging to the genus *Methanosaeta*, dominated the phylum along the entire operation period, with abundances ranging between 37.2% at day 1 and 82.2% at day 301 (Figure 6). Within the *Methanomicrobia* class, the families *Methanocorpusculaceae*, *Methanomicrobiaceae*, *Methanoregulaceae*, *Methanospirillaceae*, and *Methanosarcinaceae* represented up to 19.4% of the total reads. *Methanobacteriaceae* decreased along the operation, passing from 28.3% of the total reads at day 1 to 4.5% at day 315. *Methanofastidiosaceae* increased from day 56, representing up to 23.4% of the total reads at day 112. As shown in Figure 6, hydrogenotrophic methanogens taxa like *Methanobacterium*, *Methanobrevibacter*, and *Methanocorpusculum* decreased over time and were only marginally found at steady-state operating conditions. The relative abundance of the phyla *Altiarchaeota*, *Crenarchaeota*, and *Nanoarchaeaeota* were high during the start-up, representing up to 27.3% of the total reads, and decreased after steady-state conditions were reached (on average 1.7%, Figure S2).

## 4. Discussion

Most of the anaerobic digestion studies previously carried out were aimed at characterizing the structure and composition of microbial communities in AD during the operation in the stationary phase rather than in the start-up phase [8,35,36]. Furthermore, to the best of the authors’ knowledge, there are no studies regarding the use of the high-throughput sequencing approach to characterize the microbial populations involved in the start-up phase of a municipal WWTP anaerobic digester. In this study, the analysis of the microbial populations in a real system, susceptible to operational variations typical of real full-scale systems, was performed. Moreover, the population dynamics were analyzed during the entire operation of a full-scale system, from the start-up phase up to the stable production of methane. The digester investigated was first filled and then fed with the same WAS sampled in the Fusina WWTPs.

The steady-state condition started to develop after 2 HRTs as indicated by the progressive stabilization of the temperature, pH, and alkalinity levels. As shown in Figure 1, both the pH and alkalinity started increasing during the first 40 operation days following the digester temperature increase, before stabilizing permanently around day 80. Conversely, methane production showed a delayed behavior during the transient conditions. It started to increase only after 40 days and reached stability only after day 100, with the consumption of the VFAs accumulating during the first days of operation. In accordance with our findings, Dohdoh et al. [37] successfully applied at the lab scale the strategy to start-up the sewage sludge digestion process without external inoculum, affirming that the steady-state condition was reached after 73 days of operation.

As regards anaerobic digesters treating animal or agro-industrial waste, generally characterized by longer start-up times, Angenent et al. [25] observed the achievement of the steady state after 90 days for a digester treating swine waste, using the anaerobic sludge collected from a municipal WWTP as inoculum. Ike et al. [16] reported a 150-day startup period for a digester treating industrial food waste (coffee grounds, potato waste, beans, and tofu waste) by using an inoculum derived from a cow and pig manure treatment plant. On the contrary, Goux et al. [17] reported a start-up period of 175 days for an anaerobic digestion process carried out on plant biomass and animal effluents, without adding any external inoculum.

The achievement of the steady state in the digester was mirrored by the establishment of a microbial population with a different composition with respect to the one observed during the start-up phase. In line with the low methane production and the high VFA accumulation, over 1 HRT, a marked decrease in the specific abundance of bacteria and methanogens was observed, followed by the subsequent increase of both microbial components when the steady state was reached. In contrast with this finding, Podmirseg et al. [38] observed a dynamic archaeal community and a relatively stable bacterial community during dramatic environmental changes of a successful full-scale reactor start-up. The reduction of bacteria and methanogen abundance observed in our study during start-up phase was probably linked to the acclimatization of the microorganisms occurring in the inoculum to the new conditions of anaerobiosis in the digester and the selection of taxa with high resistance to perturbations.

**Figure 7:**
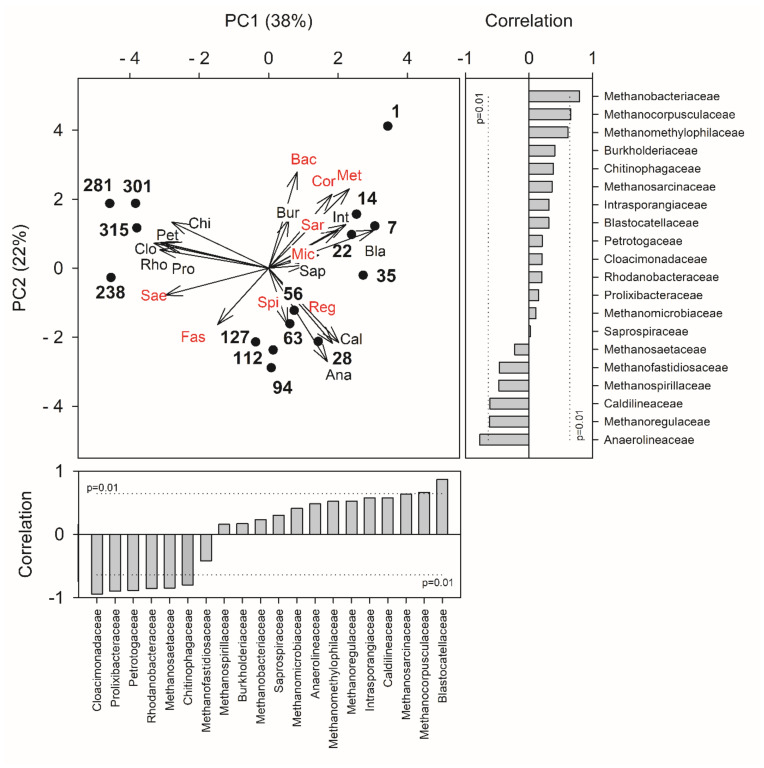
Principal components analysis biplot performed using bacterial (in black) and archaeal (in red) families (>5%) as revealed by 16S rRNA gene high-throughput sequencing. The vector length is proportional to the correlation between the corresponding parameter and the PCA axis 1 and 2. Histogram plots show the contribution of each variable (vector projection values) expressed as the correlation with the x- and y-axis (PC, Principal Component). Ana, Anaerolineaceae; Bac, Methanobacteriaceae; Bla, Blastocatellaceae; Bur, Burkholderiaceae; Cal, Caldilineaceae; Chi, Chitinophagaceae; Cor, Methanocorpusculaceae; Clo, Cloacimonadaceae; Fas, Methanofastidiosaceae; Int, Intrasporangiaceae; Met, Methanomethylophilaceae; Mic, Methanomicrobiaceae; Pet, Petrotogaceae; Pro, Prolixibacteraceae; Reg, Methanoregulaceae; Rho, Rhodanobacteraceae; Sae, Methanosaetaceae; Spi, Methanospirillaceae; Sap, Saprospiraceae; Sar, Methanosarcinaceae.

Therefore, once steady-state conditions were achieved, taxa uncommonly reported in full-scale anaerobic digesters, but found to be distinctive features in the feed sludge, were retrieved as key components of the bacterial microbiome. As shown in Figure 7, the principal component analysis (PCA) allowed recognition of the variation patterns of major microbial families passing from the start-up to the last days of operation. The first component explained most of the variance within the dataset (38%), thus discriminating between the operating days. The relative percentage of the family *Blastocatellaceae* was higher during the start-up, showing a positive correlation with the PC1 axis. In contrast, the families *Cloacimonadaceae*, *Prolixibacteraceae*, *Petrotogaceae*, *Rhodanobacteraceae*, *Methanosaetaceae*, and *Chitinophagaceae* were relatively more concentrated during the last days. Those microbiological variables negatively correlated with the PC1 axis, largely the most explicative of the dataset variability (Figure 7). PCA analysis highlighted how starting from an aerobic sludge, the imposed operating conditions promoted a microbial population evolution in the reactor, leading to the formation of two distinct clusters between the samples analyzed during the start-up and stationary phase.

In fact, families mainly composed of aerobic microorganisms like *Blastocatellaceae* and *Alphaproteobacteria* were abundant during the start-up phase but decreased over time. Under steady-state operating conditions, the concentration of total bacteria was stable and about two orders of magnitude higher than that of methanogens. The bacteroidetes phylum increased along the whole operation. *Bacteroidetes* were suggested to be important in a hydrolytic and acidogenic digester and enough to be considered as responsible for protein, fat, cellulose, and other polysaccharides degradation and fermentation during the AD process [39,40]. In particular, in this phylum, a switch between the *Saprospiraceae*, and *Prolixibacteraceae* and *Chitinophagaceae* families was observed. Indeed, members of the Saprospiraceae family are mainly aerobic so the ASVs affiliated with this phylum decreased over time while the *Prolixibacteraceae* and *Chitinophagaceae* families increased, ensuring a hydrolytic and fermentative bacterial portion in the system.

The anerobic conditions in the digester promoted the selection of an active methanogenic biomass during the start-up phase. Indeed, an increase of the specific methane production (about 0.144 ± 0.03 m^3^CH_4_/kgVSS_fed_ after 1 HRT) was observed together with an increase of methanogenic archaeal gene copies (Figure 2 and Figure 4). At the same time, the remarkable VFA consumption induced pH and alkalinity variations, favoring the achievement of the steady-state phase (Figure 1 and Figure 2). The composition of the methanogenic core in the digester was dissimilar to previous investigated start-up systems, most likely due to the different inoculum and substrate used [16,17,25]. Instead, the archaeal community was in line with the microbiome retrieved in a full-scale anaerobic digester treating sludge [8]. The *Methanosaeta* family settled in the digester already during the start-up phase (37% of the total reads) to cover most of the sequences detected in the steady-state condition (over 80% of the total reads). As is generally known, the *Methanosaeta* family had a competitive advantage with respect to *Methanosarcina* during continuous operation in the reactor fed by WAS [8]. Data obtained in this study highlighted that methanogenesis was mainly performed by acetoclastic microorganisms. The achievement of the steady state coincided with the reduction of ASVs affiliated with hydrogenotrophic taxa, namely *Methanobacterium*, *Methanobrevibacter*, and *Methanocorpusculum*. Nevertheless, the presence of *Candidatus Methanofastidiosum* in the steady-state phase indicated a potential hydrogenotrophic methanogenesis, since members of these taxa are able to consume H_2_ at a high rate only in the presence of an active methanogenic biomass [8,41,42].

**Figure 8:**
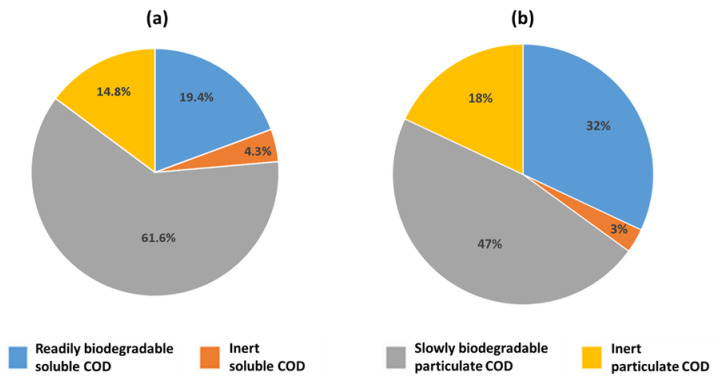
COD fractionation of the Fusina WWTP influent wastewater (**a**), compared to the typical Italian average COD fractionation (**b**). The influent inert soluble COD was estimated as 90% of the COD of the WWTP effluent. The readily biodegradable COD was calculated as the difference between the soluble COD and the inert soluble COD of the WWTP influent. The slowly biodegradable particulate COD was calculated as the difference between the BOD20 and the readily biodegradable COD of the WWTP influent. The inert particulate COD was calculated by subtracting from the total influent COD the readily biodegradable soluble COD, the inert soluble COD, and the slowly biodegradable particulate COD.

According to Dohdoh et al. [37], a successful digester start-up could be obtained using WAS as the substrate for anaerobic degradation. In addition, these results showed that the microbial composition in the digester was strongly influenced by the quality of the activated sludge used at first as the inoculum and then as daily feed. In particular, the same microbial groups closely associated with municipal WWTPs can be found in the anaerobic digester. The bacterial composition of Fusina WAS had a characteristic structure that differed in part from the typical average sludge composition reported in the literature [9]. WAS samples were collected and analyzed in four different periods in order to determine the core microbiome. Data showed no significant differences in the sludge composition along the sampling period, indicating a strong microbial core selected by the process. The most abundant sequences detected were affiliated with the *Proteobacteria* phylum, which is a typical group found in sewage sludge [9]. In contrast, the distinctive presence of facultative, anaerobic, and hydrolytic bacteria was observed in this WAS. Indeed, this group of microorganisms was not frequently reported among the major bacterial constituents in similar systems [9]. For example, Fusina sludge showed the presence of the Saprospiraceae family for about 17% of the analyzed sequences. It is well known that Saprospiraceae species play an important role in hydrolysis in different systems [9]. The abundance of this family in WAS is reflected in the composition of the digestate. In fact, the portion of *Bacteroidetes* in the digester gradually increased during its operation. This trend, combined with the selection and increase in ASVs associated with families, such as *Cloacimonadaceae* and *Petrotogaceae*, can be explained again by the nature of the feed sludge. In fact, the *Cloacimonetes* phylum has been associated with the anaerobic processes of cellulose degradation and propionate oxidation, with the latter being a fundamental step in anaerobic digestion [43]. Moreover, *Petrotogaceae* reads have been observed in reactors involved in the fermentation of complex polysaccharides [44,45]. Similarly, the Fusina WAS presented a high percentage of recalcitrant or slowly degradable COD. Such a phenomenon derives from the peculiar characteristics of the sewage entering the WWTP (Figure 8), in turn due to the particular morphology of the area of Venice and its surroundings served by the plant. The Fusina sewer network is in fact made up of long pipelines and several lifting stations, thus leading to long retention times of the sewage in the network, resulting in partial in-sewer biodegradation of the wastewater before the oxidation tank, with consequent partial consumption of readily degradable COD.

For this reason, the wastewater treated by the Fusina WWTP is characterized by a fraction of soluble degradable COD about 40% lower (Figure 8) than the typical value observed in the Italian municipal wastewaters [46]. This also forces the WWTP managers to add external readily degradable COD—in the form of acetate—during the pre-denitrification phase, to ensure the efficiency of the nitrate removal process.

At the same time, the Fusina wastewater contains high contents of biodegradable organic substance in particulate form (about 62% of the total COD). Moreover, the WWTP is not equipped with primary sedimentation tanks; therefore, all the influent COD is treated in the oxidation tank and the anaerobic digesters are fed with WAS only. Therefore, it is possible to speculate that such a unique characteristic of the treated wastewater is reflected in the WAS composition and, successively, in the digested sludge composition.

## 5. Conclusions

A successful start-up phase, carried out without adding external anaerobic inoculum, of a full-scale mesophilic digester treating activated sludge was investigated in this work for the first time. Fusina sludge used to inoculate the digester was microbially characterized several times and a typical population composition, rich in specific hydrolytic bacteria attributable to the high recalcitrant COD present in this WWTP, was found. This study showed that the microbial composition in the digester was strongly driven by the sludge used as inoculum and then as feed. Marked bacterial and archaeal successional dynamics were observed during long-term plant operation. In particular, a shift between the start-up and steady-state phase due to the acclimatization of microorganisms to the different operating conditions was observed. Despite the use of an aerobic sludge as inoculum, the methanogens present in the anaerobic niches in the sludge flocs ensured a good production of methane after the start-up phase, when the methanogenic community was enriched in acetoclastic Metanosaetaceae over time.

## Data Availability

Not applicable.

## Supplementary Materials

The following are available online at https://www.mdpi.com/article/10.3390/microorganisms9122581/s1, Figure S1: Frequency heat-map of bacteria communities at taxonomical phylum levels during digester operation. The color intensity shows the relative abundance of the different groups, Figure S2: Frequency heat map of archaeal communities at taxonomical phylum levels during digester operation. The color intensity shows the relative abundance of the different groups.
