# Supplementary material for: Microbial Community Successional Changes in a Full-Scale Mesophilic Anaerobic Digester from the Start-Up to the Steady-State Conditions
Source: Microorganisms. 2021 Dec 13;9(12):2581. doi: 10.3390/microorganisms9122581 (PMC8704592; doi:10.3390/microorganisms9122581)
Supplement: Supplementary file 1 [file microorganisms-09-02581-s001.zip › microorganisms-1469583-supplementary.pdf]

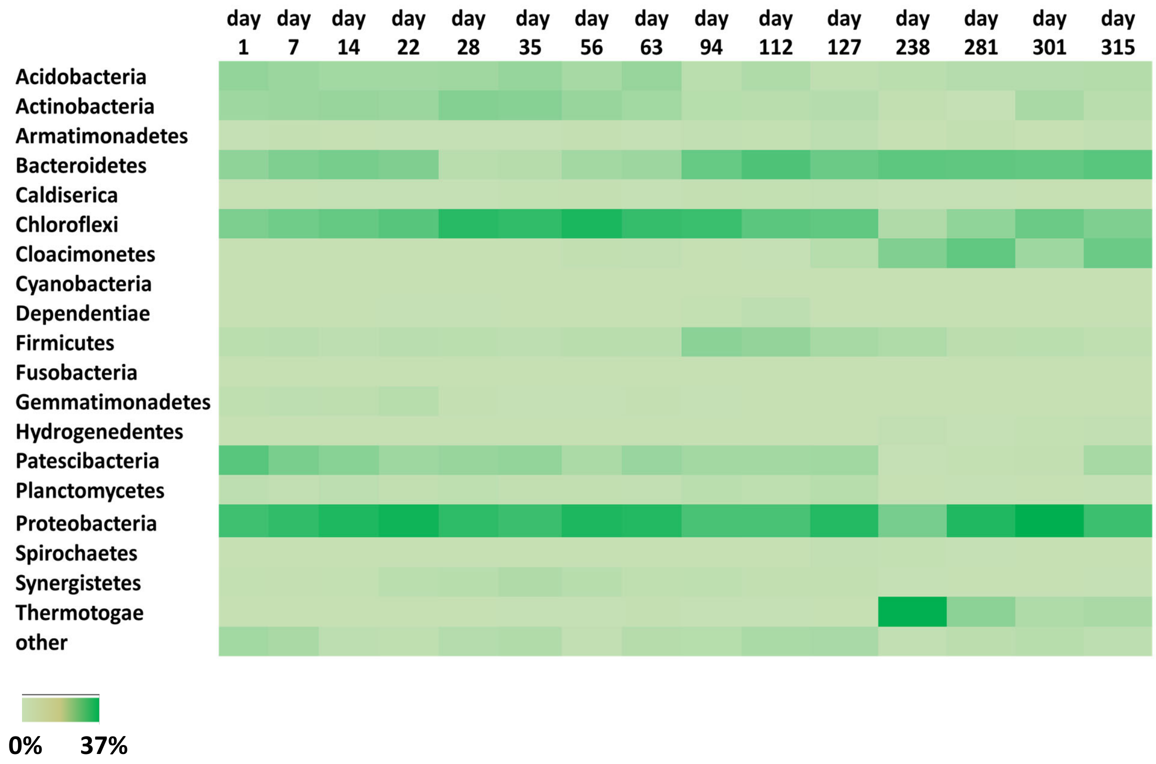

Figure S1. Frequency heat-map of bacteria communities at taxonomical phylum levels during digester operation. The colour intensity shows the relative abundance of the different groups.

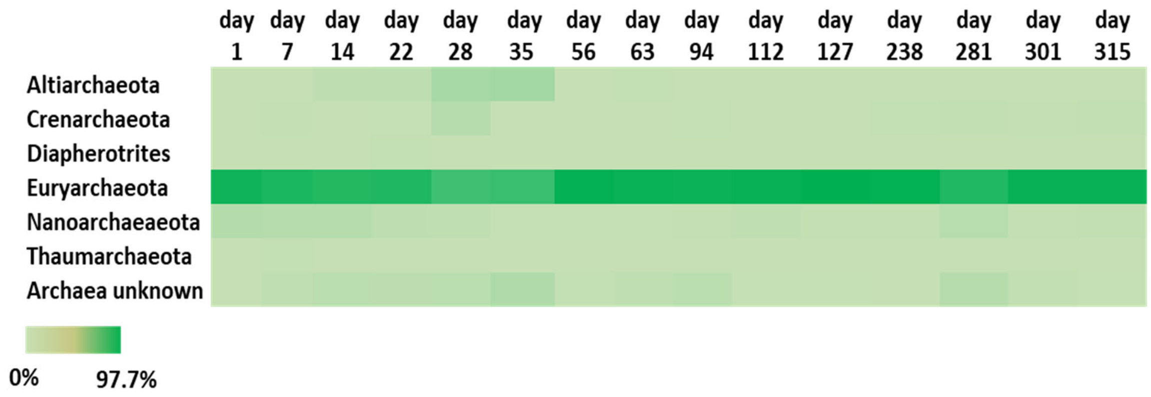

Figure S2. Frequency heat-map of archaeal communities at taxonomical phylum levels during digester operation. The colour intensity shows the relative abundance of the different groups.
